# Beyond problematic social media use and the brain: A public health and policy perspective

**DOI:** 10.1111/nyas.15409

**Published:** 2025-06-30

**Authors:** Holly Shannon, Matteo Montgomery, Alison Funk, Alireza Kamyabi, Madison Hunt, Ceinwen Pope, Kim Hellemans, Synthia Guimond

**Affiliations:** ^1^ Department of Neuroscience Carleton University Ottawa Ontario Canada; ^2^ University of Ottawa Institute of Mental Health Research at The Royal Ottawa Ontario Canada; ^3^ Vancouver Coastal Health Vancouver British Columbia Canada; ^4^ Department of Health Sciences Carleton University Ottawa Ontario Canada; ^5^ School of Population and Public Health University of British Columbia Vancouver British Columbia Canada; ^6^ Département de Psychoéducation et Psychologie Université du Québec en Outaouais Gatineau Quebec Canada; ^7^ Department of Psychiatry University of Ottawa Ottawa Ontario Canada

**Keywords:** behavioral addiction, mental health, problematic social media use, public health, social media policy

## Abstract

Growing concerns about the impact of social media on youth mental health have been a topic of particular interest. However, maladaptive patterns of use, such as problematic social media use (PSMU), do not currently have official diagnostic recognition as a possible behavioral addiction. This commentary discusses PSMU within a framework of addictive behavior and the underlying neurobiological mechanisms. In addition, opportunities to prevent and mitigate PSMU in public health policy and practice are explored, including health protection, preventive interventions, assessment and surveillance, and health promotion. This comprehensive approach incorporates learning from existing public health frameworks and parallels to behavioral and substance use addictions.

## Graphical abstract

With the rise in social media use comes concerns of problematic patterns of engagement resembling behavioral addictions. We explore the classification, debate, and underlying neurobiological mechanisms of problematic social media use (PSMU). In addition, public health frameworks to mitigate PSMU through health protection, prevention, surveillance, and health promotion are discussed.

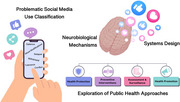

## INTRODUCTION

Over five billion people use social media platforms regularly, according to a recent global report.^1^ The average time spent using social media is increasing each year, with an average of 143 min per day in 2024 as compared to 90 min in 2012.^2^ Accompanying this heightened social media use comes growing concerns about its potential impact, particularly pertaining to mental health and especially among youth.

A recent advisory from the US Surgeon General highlights the potential harms of social media use among children and adolescents, namely, symptoms of depression and anxiety.^3^ However, according to the National Academies of Sciences, Engineering, and Medicine, there is no clear evidence of a population‐level causal relationship between the number of hours social media is used and adolescent depression.^4^ Instead, there exists a complex relationship influenced by individual differences, social contexts, and types of use.^4^, ^5^ That said, problematic social media use (PSMU), characterized by addictive and compulsive patterns of use that lead to distress or impairment, does appear to be significantly associated with higher depression and anxiety.^6^, ^7^, ^8^ This commentary will focus on considering PSMU through a behavioral addictions framework lens and will explore the potential approaches and remaining questions from this framing.

## CLASSIFYING PSMU

Both excessive engagement and adverse patterns of social media use can contribute to maladaptive behaviors characterizing PSMU.^9^, ^10^ PSMU is defined not only by frequency of use, but by measuring symptoms related to addictive disorders.^11^, ^12^, ^13^ A combination of criteria used to diagnose substance use disorders has been adopted to identify behaviors that might characterize PSMU.^14^, ^15^ For example, the Bergen Facebook Addiction Scale reflects the six core elements of addiction: (1) salience, (2) tolerance (needing to be on social media for longer periods of time), (3) mood modifications, (4) conflict, (5) withdrawal symptoms, and (6) relapse.^11^ An adapted version of this scale has been validated to measure social media platforms in general, becoming one of the most widely used tools to measure PSMU.^12^, ^16^, ^17^, ^18^ Despite the increasing recognition of PSMU as a possible behavioral addiction, it is not currently recognized as an official diagnostic term by the *Diagnostic and Statistical Manual of Mental Disorders, 5th ed*. (DSM‐5) or the *International Classification of Diseases‐11th Revision* (ICD‐11).^19^, ^20^

The DSM‐5 includes two subdivisions to the category of substance‐related and addictive disorders, consisting of substance‐related and non‐substance‐related disorders.^19^ Since non‐substance‐related disorders do not involve the consumption of a psychoactive substance, these disorders are commonly referred to as behavioral addictions. Currently, in terms of behavioral addictions, gambling disorder is the only diagnosable disorder in the DSM‐5.^19^ As part of its appendix, the DSM‐5 includes internet gaming disorder as a potential disorder for future consideration, and requests further research.^19^ Similarly, the ICD‐11 now recognizes both gambling and gaming disorders as clinically significant syndromes characterized by habitual rewarding behaviors that take priority over daily activities, interfere with personal functioning, cause distress and impairment, and persist despite negative consequences due to impaired control.^20^ Further research is required to determine if similar criteria can be applied to PSMU, as the application of classical components of addiction is currently under deliberation.^21^ It may be useful to conceptualize online‐based behavioral addictions as part of a broader spectrum of maladaptive interactive media use, similar to how substance use disorders encompass various substances under a unified framework.

Considerable debate surrounds the clinical relevance of classifying PSMU as a behavioral addiction and concerns about over‐pathologizing everyday behavior.^22^ After all, social media use is a pervasive modern behavior associated with a spectrum of outcomes ranging from beneficial to harmful. However, classifying PSMU is not meant to pathologize social media, but to define when and how it can become harmful. As with recognized behavioral addictions, PSMU must be explicitly associated with functional impairments rather than just excessive use.^23^ Understanding the development and trajectory of PSMU will provide critical insight into how to address social media through clinical and public health policy and practice.

Research has demonstrated that screen time itself is a relatively poor predictor of PSMU.^24^, ^25^ Instead, PSMU is most strongly correlated with specific patterns of behaviors on social media and underlying cognitive and mental health challenges.^6^, ^7^, ^25^, ^26^, ^27^ Certain groups may be at an increased risk of developing PSMU. For example, pre‐existing mental health symptoms and low social popularity, especially among adolescent females, have been implicated in harmful online behaviors and PSMU.^28^, ^29^ Moreover, PSMU has been linked to eating disorders, highlighting an important association to consider, as both involve the dysregulation of routine behaviors.^30^, ^31^ However, some historically marginalized populations (e.g., LGBTQIA2S+ youth) may benefit from social media due to online community connections and support.^4^ Ongoing research must identify the specific behaviors and traits that contribute to PSMU, while considering the known benefits for certain populations.

## PSMU IN THE BRAIN

A traditional addiction framework attempts to identify the underlying mechanisms that contribute to the development and maintenance of addictive behaviors. These mechanisms exist across the stages of binge/intoxication, withdrawal/negative affect, and preoccupation/anticipation, where there is a dysregulation of neural circuits in these domains.^32^, ^33^ A core feature across addictive disorders is aberrations in the reward circuitry in the brain,^33^ specifically, the mesocorticolimbic dopamine system, which includes the striatum.^34^, ^35^ To date, excessive social media use has been correlated with reductions in the gray matter volume of striatal regions.^36^, ^37^ Structural abnormalities could underlie maladaptive reward processing, as a result of the reinforcement social media provides, and, therefore, perpetuate problematic use behaviors.^37^

In addition to structural brain changes, behaviors implicated in PSMU are associated with functional changes in reward processing and inhibitory control.^27^ Sherman et al. conducted two functional magnetic resonance imaging (fMRI) studies in which adolescents and young adults interacted with a simulated social media feed.^38^, ^39^ Both providing and receiving “likes” were correlated with significant activation in ventral striatal regions like the nucleus accumbens and ventral tegmentum.^38^, ^39^ To better understand how this pattern of online socialization impacts the adolescent brain, Maza et al. conducted a longitudinal fMRI study examining habitual checking of social media.^40^ Relative to controls, participants with habitual checking behaviors had a steady increase in activation of brain regions associated with reward (i.e., ventral striatum) and cognitive control (i.e., dorsolateral prefrontal cortex).^40^ The authors postulated that this trajectory reflects an increasing sensitivity to online social rewards and difficulty in inhibitory control, similar to what is seen in substance use disorders.^40^

It is well known that highly salient rewarding events experienced during development (e.g., using addictive substances) may shape and alter the reward pathway and adult propensity to psychiatric disorders, including substance use disorders.^32^ However, little, if anything, is known about the role of early social media use on brain development, especially when use may be considered problematic.^41^, ^42^ There is an urgent need for more research to understand the neurobiological mechanisms and behaviors underlying PSMU. Specifically, larger sample sizes, varying techniques, and interdisciplinary perspectives are essential to determine how social media affects brain development, its potential links to other addictive behaviors, and the effect of social media design on neural processes.^43^ Such research is crucial in designing policy and social media platforms that promote health and well‐being.^43^

## CURRENT PUBLIC HEALTH AND POLICY ON PSMU

There exist opportunities to prevent and mitigate PSMU in public health policy and practice by incorporating lessons from existing public health frameworks and parallels with behavioral and substance use addictions.^44^, ^45^ Social media is a relatively new phenomenon where evidence, policies, and practices are actively trying to keep up with rapid technology changes and corresponding population impacts. Pathways for harm include misinformation spread, privacy and security concerns, and exposure to harmful content.^35^, ^36^, ^37^ Globally, many policy approaches addressing social media have focused on tackling potential harms, mainly privacy concerns and the spread of misinformation.^46^, ^47^, ^48^, ^49^, ^50^, ^51^, ^52^, ^53^ Examples include the Canadian Digital Charter and the Online Harms Act, and the American Children's Online Privacy Protection Act (COPPA).^49^, ^50^, ^51^ Recent moves by some countries and sectors have also focused on limiting availability for youth, such as bans in schools and requiring parental consent for youth under 16.^54^ Few policies have been in place long enough to understand their effectiveness and potential unintended consequences. Although there is increasing recognition, few policies have comprehensively focused on targeted measures for specific aspects of social media that may contribute to problematic use.^44^

A clearer understanding of how and when social media use may become problematic is necessary. This is important for informing a public health approach that weighs potential harms and benefits for the population. Other comparable public health issues, where problematic use is concerned, employ universal policies, such as limits to availability and increased sector regulation.^46^ In addition, targeted approaches such as protections for those at highest risk and addressing structural factors that perpetuate problematic use are utilized.^47^ However, the impact of social media on the population and its prominent place in society require recognition that some universal measures may not be beneficial.^48^ Limiting social media across the board may not achieve the same public health goals as comparable issues like problematic gambling or alcohol use. More research is required to examine the specific factors, behaviors, and psychological processes that may contribute to PSMU.^49^ This approach is supported by the interaction of person‐affect‐cognition‐execution (I‐PACE) model for addictive behaviors, which explains how dynamic interactions between predisposing factors, the environment, and emotional/cognitive response patterns drive habitual addictive behaviors.^50^ Recognizing varying stages from nonuse to high risk, as seen in the substance use spectrum, may be beneficial in understanding when social media use can become problematic.^51^

## INFORMING PUBLIC HEALTH AND POLICY: SOCIAL MEDIA SYSTEM DESIGN

PSMU is not merely the result of individual behaviors, but rather the outcome of systems intentionally designed to maximize user engagement.^52^, ^53^ Features like infinite scrolling, algorithmic content personalization, and gamified notifications have been engineered to capture attention and encourage habitual use.^55^ This intentional design is likely to be a strong contributor to PSMU.^52^ Parallels for other addictive disorders exist, where structural characteristics and systems design are known contributors to problematic use.^52^ For example, structural gambling factors such as short time delay between gamble and outcome, losses disguised as wins, and near‐misses are considered to shape problem gambling.^56^

While empowering individuals to adopt healthier digital habits is important, such approaches often fall short because they rely on self‐control and behavior change in a digital environment designed to exploit cognitive vulnerabilities. The automaticity and habitual nature of social media use make it difficult for individuals to consistently manage their usage, even when equipped with tools like time‐limit apps or usage trackers.^45^, ^57^ Youth may be particularly at risk of these persuasive design features due to their developing brain and limits on making good judgments, regulating emotions, and sensitivity to rewards.^24^

Addressing system design, stemming from a data business model, is a way to target the role of platform design in PSMU.^58^ With a growing public awareness of how screens, particularly social media apps, are impacting people across age groups, new opportunities have emerged for change. Interventions such as limiting addictive features, introducing design frictions like grayscale mode, and mandating ethical design standards can disrupt problematic engagement patterns and promote mindful technology use.^59^, ^60^ Ultimately, tackling PSMU lies in reshaping the systems and design elements that underpin these platforms. Such approaches align with public health principles by addressing the structural factors that may drive PSMU, rather than solely targeting individual behaviors.

## EXPLORATION OF PUBLIC HEALTH APPROACHES

**FIGURE 1:**
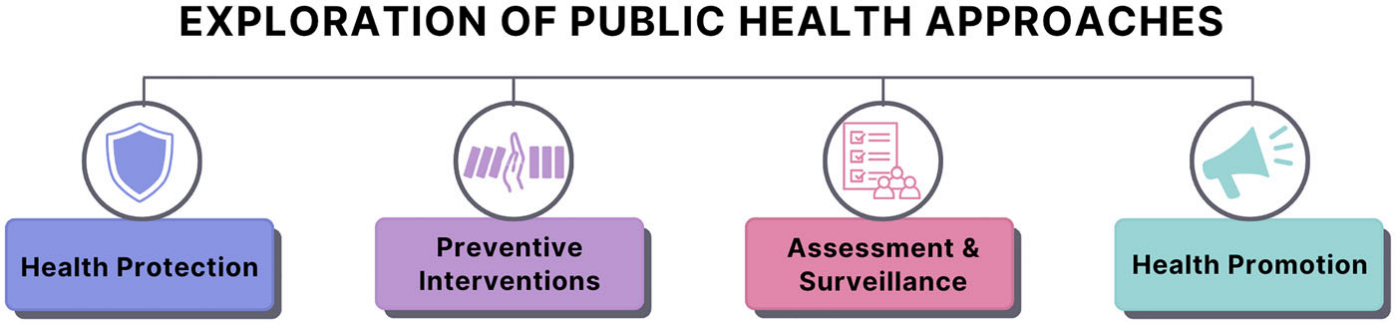
Illustration of public health approaches for exploration.

In light of the growing evidence of the negative impacts associated with PSMU and the youth mental health crisis, this paper advocates for taking a comprehensive public health approach to addressing PSMU. A public health approach involves examining the systemic and structural factors influencing social media use and determining solutions that balance the potential harms and benefits in proportion to their population impact. While PSMU presents novel challenges, existing public health frameworks addressing comparable issues, such as problematic alcohol consumption and gambling, offer valuable insights.^61^ Opportunities for action within the core public health functions of health protection, preventive interventions, assessment and surveillance, and health promotion are applied below to demonstrate the complementary actions necessary to address PSMU and safeguard public health (see Figure 1). Importantly, equity considerations, including strategies to protect groups at heightened risk of PSMU, must be central to this approach.

### Health protection

Health protection plays a critical role in mitigating harm through legislation, regulation, and enforcement aimed at creating safer systems and environments. The largely unregulated nature of social media platforms has perpetuated detrimental practices, such as addictive features and algorithmic content delivery designed to maximize user engagement. This is paired with very few limitations on availability and access, especially for users more at risk of problematic use. International examples illustrate potential health protection strategies. In Australia, legislation has been proposed to require parental consent for users under 16, along with privacy and safety regulations targeting younger populations.^62^ Policy strategies that delay the age of initial social media use are promising, as developing brains are likely to be more susceptible to PSMU.^24^ Similar strategies are used to minimize the risk of problematic gambling disorder for young people, who are similarly susceptible due to brain development. In the 2024 US Surgeon General's Advisory on Social Media and Youth Mental Health, Vivek Murthy recommended using warning labels as a disclaimer on apps.^54^, ^63^ Warning labels could illustrate the connection between PSMU and associated risks to mental health. Exploring these types of interventions may decrease the risk of PSMU for those who are most susceptible, particularly youth.

In recent years, public health organizations across North America have increasingly acknowledged the need to address the design of digital environments, which are mainly driven by commercial interests that conflict with public health goals. Addressing industry‐driven interests through regulatory actions has long been a strategy to mitigate the harms of tobacco, and there is a role for similar approaches in PSMU. Similar to the Tobacco Reporting Regulations in Canada, social media companies could be required to report on their activities and elements of their platforms that are in the interest of the public.^64^ Strengthening regulatory mechanisms to limit aspects of social media platforms that contribute to problematic use could be explored. There is a call for a paradigm shift that balances innovation with safeguards for public health, whereby technology developers hold ethical responsibility. This approach recognizes that reshaping the systems behind social media is essential to mitigate the possible negative effects.

### Preventive interventions

Preventive interventions like counseling, screening, and early detection are key components of public health.^61^ Though PSMU is not currently recognized as a behavioral addiction, developing strategies across the spectrum from primordial, primary, secondary, and tertiary prevention is likely to be necessary, as with substance‐related and other addiction disorders. This may include low‐risk social media use guidelines, screening and early intervention tools that can be used in clinical and community settings, and treatment approaches to mitigate further harms. Some clinical screening tools currently exist for PSMU, including the Problematic and Risky Internet Use Screening Scale (PRIUSS‐3) and the HEADS‐4 screening tool.^65^ Importantly, more research into risk and protective factors across the lifespan will be necessary to develop effective prevention approaches.

### Assessment and surveillance

Assessment and surveillance are critical components of the public health framework, serving as tools to monitor population health, evaluate the effectiveness of interventions, and guide evidence‐based decision‐making. In the context of addressing PSMU, assessment and surveillance play an essential role in understanding the prevalence, patterns, and consequences of digital behaviors. By providing insights into how individuals are engaging with social media, public health professionals can identify key areas for intervention and develop strategies to mitigate PSMU.

Many population health surveys focus on the quantity of use and have not yet integrated other indicators of problematic use.^66^ Public health must prioritize incorporating validated tools in population‐level surveillance. Existing clinical screening tools like the PRIUSS‐3 and HEADS‐4 could be adapted for population‐level use. Innovative methods, such as passive data collection through digital applications or anonymized tracking systems, can also help capture more accurate usage patterns while respecting user privacy.^66^ Strengthening opportunities for data sharing between social media platforms and public health researchers could allow for a comprehensive evaluation of usage patterns and their impacts.^67^

### Health promotion: Creating supportive environments and healthy public policy

The environments in which we live can change the likelihood that we take part in behaviors such as PSMU. Effective public health strategies to limit the local availability of legal psychoactive substances like alcohol or tobacco through healthy public policy are less applicable to social media due to the nature of its accessibility to individuals. However, there are some strategies that can be applied to help create healthier environments. For example, schools have been identified as a key setting for screen use interventions due to the amount of time young people spend there. Policies limiting the use of smartphones in schools or blocking the use of social media apps can create environments that foster increased in‐person social connection, encourage learning, and support long‐term healthy patterns of use.^68^ However, it is still unclear whether uniform bans are an effective or acceptable intervention.^68^ PSMU is associated with weaker social connections and relationships, therefore, the provision of high‐quality opportunities for social connection in and outside of the classroom may help buffer the impacts of problematic use.^69^ Local government and school policies, community and social planning, and organizational policies and practices can enable more opportunities for social connection. Examples could include extracurricular activities like school gardens, inclusive sports and recreation, music, arts, and drama, volunteering, and creating spaces for outdoor play.

In the home environment, many family media plans include screen‐free zones, such as the child's bedroom or the kitchen table, providing more opportunities for balanced tech use and family connection. Social‐environmental factors like access to the internet, attitude toward usage, parental media‐use patterns, permissive social culture, and gaming industry influence the likelihood of an individual to experience PSMU and may be levers for more supportive policy change.^66^, ^70^, ^71^ Recent trends toward opening screen‐free cafes and public spaces also indicate a need for creating environments that support in‐person rather than online social connections.^72^ Changing these environments supports a shift in social norms around the way that screens and social media are used in our society.

### Health promotion: Reorienting health services

Families often turn to trusted healthcare providers for information about their child's nutrition, sleep, physical activity, and other health behaviors. Healthcare providers can play a large role in promoting positive relationships with tech and social media among their patients by sharing best practices, information about the potential benefits and risks of social media/screen use, signs of PSMU, and how to establish healthy tech habits at home.^66^ Social prescribing is an emerging tool that could be adapted to provide alternatives to social media use or integrate targeted strategies, such as through prescribing family media plans.

### Health promotion: Strengthening community action

PSMU has been an area of concern for parents for years.^72^ As a result, grassroots movements across Canada and globally (typically involving parents of young children) have taken it upon themselves to create initiatives to protect their children from online harms and problematic behaviors and to promote digital literacy at home. Some examples include organizations like Mothers Against Media Addiction (MAMA), Unplugged Canada, and Anxious Generation.^72^, ^73^, ^74^, ^75^

Similarly, schools often hear questions from parents about what the health recommendations are for screen and social media use (e.g., recommended screen time limits, recommended age of receiving a first cell phone or starting social media use, and the health impacts of social media use). Public health has an important role in providing health evidence to schools and teachers that can inform school policies, foster supportive school environments, and provide guidance for families.

### Health promotion: Developing personal skills

Traditional approaches for developing personal skills focus on empowering individuals with knowledge and skills around the potential harms of social media and strategies to address them.^66^ These range from active nudges, requiring user engagement, to passive nudges, which subtly influence behavior without conscious effort. While both approaches have value, passive nudges rooted in system design changes provide a sustainable solution for addressing ingrained smartphone habits.

Active nudges, such as time‐limit apps and planning prompts, encourage users to actively monitor and control their digital consumption. Tools like Apple's Screen Time, which tracks usage and enforces app time limits, have become widely accessible and effective at increasing digital self‐awareness. However, research shows that these tools often fall short in the long term, as many users struggle to sustain self‐control or take action beyond passive awareness of their usage habits.^57^, ^76^ This limitation underscores the challenge of relying on user discipline to disrupt habitual behaviors.

Passive digital nudges alter key design elements of smartphones and social media apps that are intentionally engineered to capture attention. Such strategies subtly reduce the hedonic value of engagement with social media apps without requiring active monitoring or intervention.^77^ Examples include changing the screen to grayscale mode, which removes the vibrant colors of smartphone interfaces to reduce the visual appeal and aesthetic engagement hedonic value, interrupting habitual interactions.^77^ These system‐level changes foster more mindful technology use while alleviating the burden of self regulation.^60^

By using both passive and active nudges in public health messaging around PSMU, individuals can be empowered to build healthier digital habits. These strategies can be shared with children and youth in educational settings or with parents and caregivers. Harvard's Center for Digital Thriving has been piloting a series of innovative lesson plans that introduce “addictive” strategies that social media companies use to capture attention, to increase motivation about having control over your own social media use.^78^ This approach aligns with broader public health goals to create supportive environments, enabling sustainable behavior change at both individual and societal levels.

## CONCLUSIONS

There is an increasing demand to continue studying the impacts of PSMU to address public health concerns and inform policy. While there are many forms of digital media available, as well as other theoretical and critical perspectives, this paper specifically centers around considering PSMU within the addictions framework. However, a broader conceptualization using other models and considering the interplay between individual, situational, and structural characteristics is still necessary.^15^, ^53^ Research regarding specific behaviors and social contexts online can aid in considering social media use as a spectrum, where use can range from beneficial to problematic. Addressing the issue of PSMU requires a comprehensive approach, including accountability from social media platforms and efforts focused on systems design. To prevent and mitigate PSMU, we suggest public health and policy opportunities for exploration drawing on parallels to behavioral and substance use addictions within health protection, preventive interventions, assessment and surveillance, and health promotion.

## AUTHOR CONTRIBUTIONS

**Holly Shannon**: Conceptualization; writing–original draft; writing–review and editing. **Matteo Montgomery, Alison Funk, Alireza Kamyabi**: writing–original draft; writing–review and editing. **Madison Hunt**: writing–original draft. **Ceinwen Pope, Kim Hellemans, Synthia Guimond**: writing–review and editing.

## COMPETING INTERESTS

The authors declare the following financial interests/personal relationships, which may be considered as potential competing interests: S.G. received financial compensation for consulting services from Boehringer Ingelheim (Canada) Ltd.

## PEER REVIEW

The peer review history for this article is available at https://publons.com/publon/10.1111/nyas.15409.
